# A Case of Heart-Filaria in a Dog

**Published:** 1880-01

**Authors:** H. E. Earl


### ART. IV.—A CASE OF HEART-FILARIA IN A DOG.

BY H. E. EARL, D. V. S.

A LARGE sized mongrel cur, who had been under observation for over a year, was noticed during that time to be failing, without presenting any such definite symptoms as would have warranted special treatment. He was sometimes noticed to be unnaturally sleepy, at others to be fretful, though never vicious, and to have a capricious appetite. He was anscultated, but nothing abnormal observed. After these symptoms had continued with considerable variation in their intensity for the period mentioned, the animal, apparently as well as usual an hour before, was found dead one forenoon.

I made the *post mortem* immediately, and examining all the organs, excepting the brain and spinal cord, found them to be structurally healthy.

On opening the *Right Auricle*, a coil of long, thin, *round worms* escaped with the blood, which was found not coagulated. On disentangling the coil it was found to consist of four individual Filaria; the largest measured *fourteen* inches in length, the others ranged between eight and ten inches. They were all in active motion, and were transferred to an alcoholic preservative fluid after a few minutes.

The large blood vessels showed no parasites, the smaller ones were not examined, the four Filaria in question were the only ones found, and three of these, including the largest, I herewith send to the editor of THE ARCHIVES for his private museum.*

I have been induced to present these brief notes of a disorder, which, however, the researches of Swinhoe, Cobbold and others have shown it to be common in China and Japan; to my knowledge no instance has been published in the journals as occurring in Europe or America. Possibly the occurrence of Filaria in the dog's heart is more frequent than is suspected, for, from the conditions of parasitic development, one would infer that where a single such case is observed, others of the kind must occur unnoticed.

* Found to be *Filaria immitis* (Leidy).—EDITOR.

